# The impact of innate immunity on malaria parasite infection dynamics in rodent models

**DOI:** 10.3389/fimmu.2023.1171176

**Published:** 2023-08-14

**Authors:** Alejandra Herbert Mainero, Philip J. Spence, Sarah E. Reece, Tsukushi Kamiya

**Affiliations:** ^1^ Institute of Ecology and Evolution, School of Biological Sciences, University of Edinburgh, Edinburgh, United Kingdom; ^2^ Institute of Immunology and Infection Research, School of Biological Sciences, University of Edinburgh, Edinburgh, United Kingdom; ^3^ Centre for Interdisciplinary Research in Biology, Collège de France, Paris, France; ^4^ HRB, National University of Ireland Galway, Galway, Ireland

**Keywords:** plasmodium, meta-analysis, within-host dynamics, innate immunity, rodent malaria

## Abstract

Decades of research have probed the molecular and cellular mechanisms that control the immune response to malaria. Yet many studies offer conflicting results on the functional impact of innate immunity for controlling parasite replication early in infection. We conduct a meta-analysis to seek consensus on the effect of innate immunity on parasite replication, examining three different species of rodent malaria parasite. Screening published studies that span four decades of research we collate, curate, and statistically analyze infection dynamics in immune-deficient or -augmented mice to identify and quantify general trends and reveal sources of disagreement among studies. Additionally, we estimate whether host factors or experimental methodology shape the impact of immune perturbations on parasite burden. First, we detected meta-analytic mean effect sizes (absolute Cohen’s h) for the difference in parasite burden between treatment and control groups ranging from 0.1475 to 0.2321 across parasite species. This range is considered a small effect size and translates to a modest change in parasitaemia of roughly 7-12% on average at the peak of infection. Second, we reveal that variation across studies using *P. chabaudi* or *P. yoelii* is best explained by stochasticity (due to small sample sizes) rather than by host factors or experimental design. Third, we find that for *P. berghei* the impact of immune perturbation is increased when young or female mice are used and is greatest when effector molecules (as opposed to upstream signalling molecules) are disrupted (up to an 18% difference in peak parasitaemia). Finally, we find little evidence of publication bias suggesting that our results are robust. The small effect sizes we observe, across three parasite species, following experimental perturbations of the innate immune system may be explained by redundancy in a complex biological system or by incomplete (or inappropriate) data reporting for meta-analysis. Alternatively, our findings might indicate a need to re-evaluate the efficiency with which innate immunity controls parasite replication early in infection. Testing these hypotheses is necessary to translate understanding from model systems to human malaria.

## 1 Introduction

The immune response plays a pivotal role in shaping the fate of infectious agents and host health outcomes (1). Innate immune effectors can block invasion, and directly kill pathogens or the host cells that they reside within (e.g., via complement, phagocytosis or ROS). For naïve hosts that have no previous exposure to malaria parasites, the innate immune system triggers a rapid effector response that is thought to provide early control of pathogen replication by removing infected red blood cells (RBC) as well as short-lived extracellular parasites known as merozoites (2, 3). This acute phase response is characterized by a pro-inflammatory environment with elevated concentrations of TNF and IFNγ, which can arrest parasite development and kill infected RBC, potentially ‘buying time’ for the development of adaptive immune responses, which are essential for parasite clearance (4). However, establishing a causal link between specific innate effector mechanisms and host control of parasite replication *in vivo* has proved challenging (5). This is largely because innate effector molecules are pleiotropic (e.g., TNF and IFNγ can also suppress erythropoiesis to reduce red cell availability) (6, 7) and because functional redundancy is a cardinal feature of the innate immune system. Consequently, it is difficult to find consensus on the extent to which innate immunity controls parasite replication and under what circumstances. To address this, we conducted a systematic and quantitative synthesis of the primary literature to ask how experimental manipulations of the innate immune system impact the dynamics of parasite replication in rodent models of malaria.

Following the discovery of rodent malaria parasites in the 1940s, model systems have been developed to provide insight into all aspects of host-parasite interactions, including mechanistic knowledge of the immune response to infection (8, 9). Rodent models offer a balanced compromise between manipulability and within-host ecological realism when compared to human infections and *in vitro* systems. Specifically, although studying human malaria offers greater clinical translational value, this is tempered by the difficulty of controlling for confounding factors in natural infections and an inability to manipulate the immune system *in vivo* (10). At the opposite end of the spectrum, *in vitro* model systems offer freedom of manipulation to probe molecular and cellular mechanisms in detail but lack realistic within-host processes that regulate dynamic immune responses (11). Thus, despite important differences to human malaria and the focus on inbred host strains, rodent models can provide general insight into how host immunity controls acute infection (12).

Many rodent malaria studies record an aspect of parasite replication (most commonly parasitaemia, which represents the proportion of infected RBC) following an experimental perturbation of the innate immune system. For example, approaches to study the impact of NOS2 (an enzyme involved in producing a reactive free radical) include infecting genetically attenuated mice (13) or mice treated with a small inhibitor drug such as aminoguanidine (14). Such experiments have been conducted on a variety of inbred host genetic backgrounds and using evolutionarily divergent parasite species (i.e., *Plasmodium chabaudi*, *P. yoelii* and *P. berghei*) that offer diverse phenotypes. For example, *P. chabaudi* is a model of chronic recrudescing infection whereas *P. berghei* can cause experimental cerebral malaria leading to rapid host mortality (15). Furthermore, these studies also vary in experimental design such as the route and dose of infection. We take advantage of the diversity of these rodent models to collate, curate and statistically analyze infection dynamics where aspects of innate immunity are manipulated, and take a meta-analytic approach to identify and quantify consensus and sources of disagreement among published studies. We then apply meta-regression models to estimate whether host factors (e.g., genetic background, age and sex), methodology (including adoptive transfer and surgery) or the statistical power of individual studies can shape the observed impact of immune perturbations on parasite burden (16).

Meta-analyses quantitatively synthesize results from multiple studies using the standard effect size to reveal general patterns in the literature (17). By pooling independent results from multiple studies, meta-analyses boost statistical power (18) and can identify sources of variability across studies to provide biological and epistemological insight (19). Moreover, meta-analyses present a more reproducible and transparent alternative to traditional narrative reviews because conclusions emerge from a statistical synthesis of systematically curated literature (20). Our meta-analysis reveals a small average effect size of experimental manipulation of the innate immune system, which equates to a 7 to 12% change in parasitaemia between control and treatment groups at the peak of infection. Meta-regression models reveal that variations in infection dynamics among studies that use *P. chabaudi* or *P. yoelii* are likely explained by stochasticity (due to small sample sizes) rather than by the effects of experimental perturbations. However, in the *P. berghei* model, experimental perturbations have a bigger impact in young or female mice, and an 18% difference in peak parasitaemia can be observed between control and treatment groups when effector molecules (rather than upstream signalling networks) are disrupted.

## 2 Methods

### 2.1 Literature search and eligibility screening

**Figure 1:**
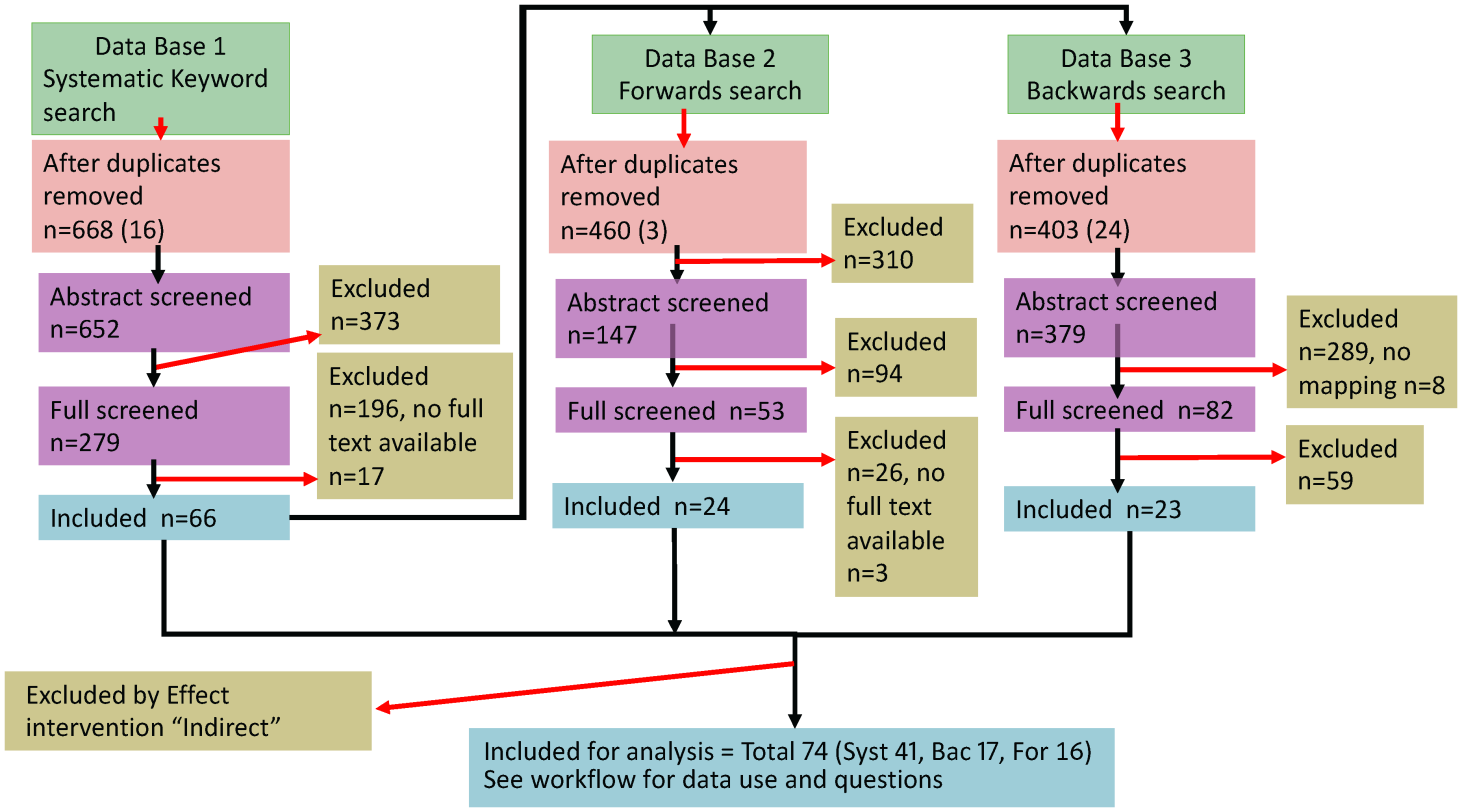
PRISMA flowchart (22) of the literature search and data extraction. Our complete dataset included 74 articles for analysis of innate immune perturbations (see **Supplementary References**). Box colours indicate numbers of articles for the literature search (green), screened (pink and purple), included (blue) and excluded studies (yellow), with the number of articles that were removed (red arrows). The following abbreviations refer to: n, number of articles; Syst, Keyword search; Bac, Backwards; For, Forwards.

Our dataset consists of published articles reporting experimental manipulations of the innate immune system in inbred mice infected with rodent malaria parasites; importantly, this only includes studies that directly manipulate the innate arm of the immune system and excludes those that might have an indirect effect (see **Supplementary Table 1**). We identified relevant articles with systematic searches of keywords as well as forward and backward citation searches. For our keyword search, we used the following keywords – innate immunity AND (*Plasmodium* OR rodent malaria) NOT mosquito* NOT human – in four databases (Pubmed, Scopus, Jstor, WoS) in January 2020. We filtered for research articles only and applied no restriction in the year of publication. We screened each article for inclusion in the dataset by first removing duplicates among searches, and then assessed the title and abstract of each article to check the experimental design and results consider a relevant topic. We then assessed the full content (where the full article was available) and retained articles that met the set of criteria based on PICO-defined elements (21). We also selected the ten oldest and newest articles that met the criteria for inclusion from the keyword search and conducted forward and backward citation searches to identify and screen relevant articles that are cited by and citing each focal article. We followed the PRISMA ECOEVO 2021 guidelines for reporting systematic reviews and meta-analyses (**Figure 1**) (22). Overall, from an initial pool of 1507 articles (668 keyword, 460 forward and 403 backward searches), 74 articles (41, 16 and 17 from the keyword, forward and backward searches, respectively) met the criteria to be included in the dataset for analysis (see **Supplementary Table 1** and **Supplementary References**).

### 2.2 Data extraction

Our study focuses on analysing patterns of parasite replication under different innate immune perturbations in rodent malaria infections. The best metric for parasite burden is density (i.e., the number of parasites per μl of blood) but this is rarely reported so instead we extracted parasitaemia (the proportion, or percentage, of infected RBC), and we required studies to report multiple temporally spaced samples for both control and treatment groups. We only included infections initiated via direct blood challenge (i.e., intraperitoneal or intravenous injection of blood-stage parasites) to specifically examine the impact of innate immunity on asexual replication during the pathogenic blood cycle. Mosquito-transmitted infections were omitted because the number of parasites that egress from the liver to initiate the blood cycle cannot be standardised or accurately estimated, which is likely to confound the analysis of infection dynamics. We focused on the acute phase of infection to minimise the influence of other host factors (e.g., RBC availability) that can mask the direct impact of the immune response on parasitaemia. Thus, we include data up to the peak of infection for *P. chabaudi* and non-lethal *P. yoelii*, which we define as the day of infection that either the control or treatment group reaches its maximum reported parasitaemia. On the other hand, we include all data for *P. berghei* and lethal *P. yoelii* because these infections are usually terminated before reaching peak parasitaemia. We analysed the four infection groups (i.e., *P. chabaudi*, non-lethal and lethal *P. yoelii*, and *P. berghei*) separately because parasite multiplication rate (and hence infection dynamics) differs markedly between them. When articles included multiple experiments, each suitable experiment was included individually. All data points from figures were extracted using Datathief III v1.7 (2015). AHM conducted all data extraction and TK validated data points. Classification of the immune covariates was determined by AHM and PJS. The full datasets used in our analyses are available in **Supplementary Data 1**.

### 2.3 Effect size – Cohen’s h

We used Cohen’s h, a standardized effect size, to quantify the difference between proportions (23). We calculated Cohen’s h for each study as the arcsine-transformation of the difference in parasitaemia in the control group compared to that in the treatment group (24). Our study includes experimental manipulations that are expected to augment [e.g., injecting recombinant IFNγ (25)] or attenuate [e.g., depleting phagocytes (26)] innate immunity, which could retard or facilitate parasite replication, respectively. We, therefore, ignore directionality (27) and use the absolute effect size in all analyses. Put another way, our estimates of Cohen’s h measure the difference between control and treatment groups but do not indicate which group has a higher parasitaemia. For ease of interpretation, we back-transform the meta-analytic mean (conditional upon the control group) to provide an estimate of the overall effect size in terms of the change in parasitaemia at the peak of infection.

### 2.4 Analysis

#### 2.4.2 Meta-analysis

We used mixed-effects meta-analytical models and all analyses were conducted in R 4.0.2 (28). We used the rma.mv function, from the metafor package (29), applying the cluster robust var-cov sandwich-type estimator to adjust for small samples and dependent structure. To account for repeated measurements due to multiple effect sizes collected from the same subjects (infections) over time, we included experiment identity as a random effect. We tested for publication bias (whether the published effect sizes were systematically skewed with respect to the overall mean) in our meta-analysis using funnel plots, regression tests and rank tests of the raw effect size. We report heterogeneity among studies as the I^2^ statistic (i.e., as the percentage of the variance between effect sizes that cannot be attributed to sampling error due to small sample sizes) (30).

#### 2.4.3 Meta-regression

Only the *P. berghei* dataset revealed residual heterogeneity (i.e., variation associated with immune perturbations that cannot be explained by small sample sizes), which allowed us to conduct meta-regression analyses to explore sources of this variation. Moderator information was only available for subsets of data so we carried out a series of univariate meta-regression analyses to examine the moderating effects of host, immunological and experimental design factors on the impacts of immune interventions. Univariate models proved the most appropriate approach due to missing data and/or small sample sizes (**Supplementary Table 2**) whilst noting that some meta-regressions involve unbalanced samples which may confound the results (16). Meta-regression analysis was performed when the dataset contained more than ten data points. The studied moderators are as follows:

- Host factors
- ○Genetic background (C57BL/6 or other)
- ○Age (centred median age reported in weeks; 5.5 to 11 weeks)
- ○Sex (female, male or mixed/unreported)
- Immunological factors (i.e., the target of experimental manipulation)
- ○Cell lineage (myeloid cells, lymphoid cells or both)
- ○Cytokines/chemokines or their receptors
- ○Effector function (inflammatory, regulatory or involved in control of cell trafficking)
- ○Position in signalling network (receptor to transcription factor (termed input) or downstream targets of transcription factors (termed output))
- Experimental design factors
- ○Sampling regime (day post-infection)
- ○Route of infection (intraperitoneal or intravenous injection of parasites)
- ○Infecting dose (number of parasitised RBC inoculated)
- ○Method of immune manipulation (genetic modification (GM), drug administration, surgery, cell transfer or mixed (any combination of these four categories))

Host genetic background was evaluated as a binary variable (C57BL/6 or other) because most studies used a single strain (i.e., C57BL/6). By pooling all other strains we prevented sample sizes from being disproportionately skewed but precluded a full comparison across genetic backgrounds. Also, not all studies reported the sex of the mice used in their *in vivo* experiments; we therefore pooled these studies with those that explicitly stated they used both sexes. This allowed us to place each experiment into one of three non-overlapping categories – female only, male only, or mixed/unreported.

Examining immunological moderators was inevitably difficult because molecules or pathways that often have pleiotropic effects required categorisation. We, therefore, approached this task as follows: if the experimental manipulation only affected cells that derive from common myeloid or common lymphoid progenitors (such as monocytes or natural killer cells), studies were classified as ‘myeloid’ or ‘lymphoid’, respectively. In cases where cell types derived from both lineages could reasonably be expected to be modified (e.g., IFNα receptor knock-out mice), studies were classified as ‘both’. Studies where cytokine or chemokine signalling cascades were targeted were categorised according to whether they manipulated the chemical messenger (‘cytokine/chemokine’) or the ‘receptor’. We next categorised studies according to whether they modified a pathway that could directly trigger or amplify inflammation (such as TNF), regulate the host response (such as lymphotoxin) or control the ‘trafficking’ or migration of immune cells (such as CXCL10). This allowed us to ask whether experiments that target the ‘inflammatory’ response have bigger effect sizes since these mechanisms of host defence are frequently cited as providing early control of parasite replication. Note that ‘regulatory’ does not necessarily mean suppressive in this context. Finally, we examined whether the position of a molecule within a signalling network had a measurable impact on effect size. Here, we simply categorised studies according to whether the experimental manipulation would alter signalling cascades before (‘input’) or after (‘output’) the induction of transcription factors (or their binding to target DNA sequences). We hypothesised that there would be greater redundancy in upstream signalling molecules (e.g., MAP kinases) than effector molecules (such as death receptors).

Full details of how each study was used to assess the moderating effects of the host, immunological and experimental design factors can be found in **Supplementary Data 1**. We conducted model comparisons using the Akaike Information Criteria corrected for small sample size (AICc) and log-likelihood ratio tests (LRT) and tested for differences between levels of a given moderator using linear hypothesis tests (29).

## 3 Results

### 3.1 Overview of study design

Our meta-analysis began with a systematic literature search. We then assessed the suitability of individual articles for inclusion before data were extracted from suitable studies and effect sizes calculated (**Figure 1**). The first phase of analysis was to calculate the overall effect size (i.e., meta-analytic mean) for each parasite species using a random-effects meta-analysis. We then estimated heterogeneity across studies to identify potential sources of variation that cannot be attributed to differences in sample size (and which may instead be explained by moderators relating to host factors or experimental design). The second phase of analysis was applied to *P. berghei*, which was the only species for which we detected non-zero heterogeneity. Here we explored the effect of biological and methodological moderators using a series of univariate meta-regressions. Finally, we assessed publication bias graphically and using statistical tests.

### 3.2 Overall effect size

**Figure 2:**
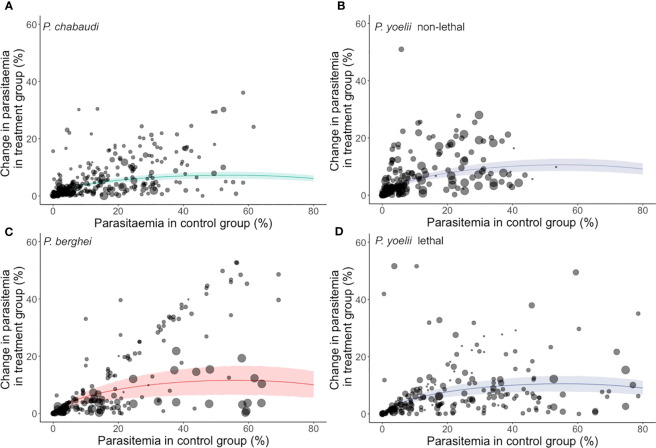
The predicted mean impact of innate immune perturbations from the meta-analytic model. The impact is expressed as the absolute difference in peak parasitaemia in a treatment relative to control group. Note that these plots do not indicate directionality, and so do not indicate that treatment groups have a higher parasitaemia than controls (or vice versa). Instead, they simply illustrate the size of the difference between treatment and control groups. Each panel refers to a different model system: **(A)** *P. chabaudi*, **(B)** *P. yoelii* non-lethal, **(C)** *P. berghei* and **(D)** *P. yoelii* lethal. Each data point represents experimental sampling points (the size of each dot indicates sample size) and the regression lines and 95% confidence intervals are shown.

Our meta-analysis found consistent effects of innate immune perturbations on parasite growth dynamics across all three *Plasmodium* species investigated (**Figure 2**). The overall effect sizes (i.e., the absolute meta-analytic mean Cohen’s h) were 0.1475, 0.2204, 0.2158 and 0.2321 for *P. chabaudi*, *P. yoelii* lethal, *P. yoelii* non-lethal and *P. berghei*, respectively. These effect sizes are modest as Cohen’s h of 0.2 and 0.5 are considered a small and medium effect, respectively (23). Back-transforming these effect sizes translates to a change in peak parasitaemia between treatment and control groups of roughly 7 to 12% (7.25%, 10.99%, 10.76% and 11.57% for *P. chabaudi*, *P. yoelii* lethal, *P. yoelii* non-lethal and *P. berghei*, respectively). Like Cohen’s h, these back transformations are agnostic of the direction of the effects (i.e., they do not report whether parasitaemia was increased or decreased in treatment groups relative to their controls).

### 3.3 Heterogeneity

We detected very low heterogeneity in the reported effect sizes for *P. chabaudi*, *P. yoelii* lethal and *P. yoelii* non-lethal (I^2^_[total]_ = 0.99%, 1.38% and 1.84%, respectively), which is consistent with the notion that random variation due to small sample sizes is responsible for the observed variability (rather than host factors or experimental approaches). In contrast, the *P. berghei* dataset contained comparatively higher heterogeneity (I^2^_[total]_ = 10.17%), suggesting that factors beyond stochasticity may underpin this variation. We, therefore, probed for potential sources of variation using a series of univariate meta-regressions, which we report in the following sections (**Table 1**).

**Table 1:** Comparisons of univariate meta-regression models against the null model, testing the effect of moderators in *P. berghei* infections.

Category	Moderator	Delta AICc	LRT	p-value	N
a) Host factors	Genetic background	0.3484	1.7165	0.1901	31
	Age	5.5859	7.6944	0.0055	20
	Sex	7.5026	11.6997	0.0029	32
b) Immunological factors	Cell lineage	2.4257	1.7713	0.4124	31
	Cytokines/chemokines or their receptors	3.8071	0.0000	1.0000	11
	Effector function	3.4694	0.7343	0.6927	29
	Position in signalling network	13.3607	15.4642	<.0001	24
c) Experimental design factors	Sampling regime	1.4336	3.5208	0.0606	31
	Route of infection	2.0703	0.0252	0.8738	28
	Infecting dose	0.4778	1.6094	0.2046	31
	Method of immune manipulation	5.6903	9.9000	0.0071	29

Delta AICc, LRT and N refer to differences in Akaike information criteria with correction, likelihood ratio test and the number of studies, respectively.

#### 3.3.1 Effect of host traits in *Plasmodium berghei* infections

**Figure 3:**
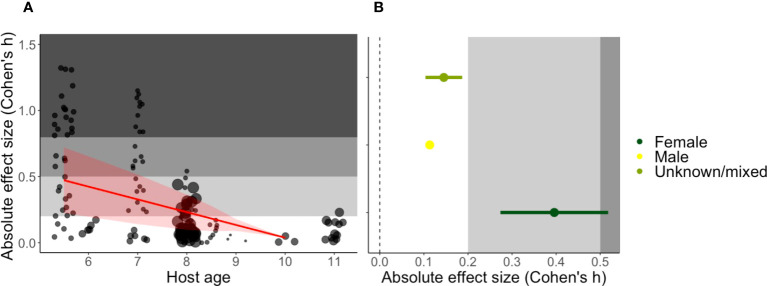
Predicted impact of host **(A)** age in weeks and **(B)** sex on the absolute Cohen’s h effect size. **(A)** The regression line is shown in red with a 95% confidence band, and the points represent studies, with point size reflecting the sample size. **(B)** Mean absolute effect ± SEs in three sex categories (female only N = 7, male only N = 1 and mixed/unreported N = 23). The grey shading denotes the reference for Cohen’s effect size bands; small (0.2) and medium (0.5) (23).

We found that the impact of innate immune perturbations declines with host age (estimate = -0.1000, SE = 0.0343, t-value = -2.9163, p-value = 0.0092, **Figure 3**). For young mice (at 5.5 weeks) the estimated absolute Cohen’s h was 0.473, which tends toward a medium effect. This constitutes up to a maximum change in peak parasitaemia of ~18% between treatment and control groups. Furthermore, studies that used only female mice exhibited a stronger impact of immune interventions than studies involving both sexes (or studies that did not report sex) (**Figure 3** and **Table 1**). The average absolute Cohen’s h among female mice was 0.396, which is considered a small to medium effect (23) and translates to a maximum difference of ~16% in peak parasitaemia. However, the lack of experiments using only male mice (N=1) precludes a direct comparison between sexes. Furthermore, the majority of studies (~60%) used a single strain (C57BL/6), likely due to the historical use of C57BL/6 mice for the generation of transgenic lines to interrogate immune cell function. This precludes a detailed analysis of the influence of host genetics (i.e., strain) but we did not find evidence that C57BL/6 mice differed from the average of all other strains combined (**Table 1**).

#### 3.3.2 Effect of targeting different aspects of innate immunity in *Plasmodium berghei* infections

**Figure 4:**
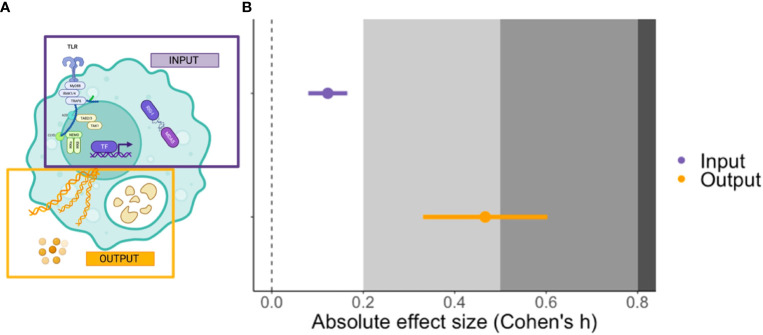
The cartoon (left, **A**) illustrates the relative position in an immune signalling network of a target of experimental perturbation: input (purple) refers to factors with an upstream signalling position (e.g., pattern recognition receptors (PRR) and transcription factors) whereas output (orange) refers to downstream effectors (e.g., cytokines, nitric oxide and proteases). The plot (right, **B**) shows the mean and ± SE effect size for input and output interventions in the *P. berghei* model. The grey shading denotes the reference for Cohen’s effect size bands; small (0.2), medium (0.5) and large (0.8) (23). The cartoon was created using BioRender.com.

Out of the four moderators examined that relate to aspects of innate immunity, only the position of the target within a signalling network revealed a significant impact, with interventions that interfere with output (i.e., events downstream of transcription factor signalling) having a significantly larger effect than those modifying input signals (receptor to transcription factor) F(1,22) = 16.8373, p-value < 0.001, **Figure 4**). The average absolute Cohen’s h for the output was 0.466, which tends towards a medium effect (23) and translates to a ~18% difference in peak parasitaemia between treatment and control groups. Other moderators did not significantly explain heterogeneity in the meta-analytic mean and these included: the cell lineage being manipulated (myeloid versus lymphoid); whether interventions targeted cytokines or their receptors; and whether effector molecules had inflammatory versus regulatory functions (**Table 1**).

#### 3.3.3 Effect of methodology in *Plasmodium berghei* infections

**Figure 5:**
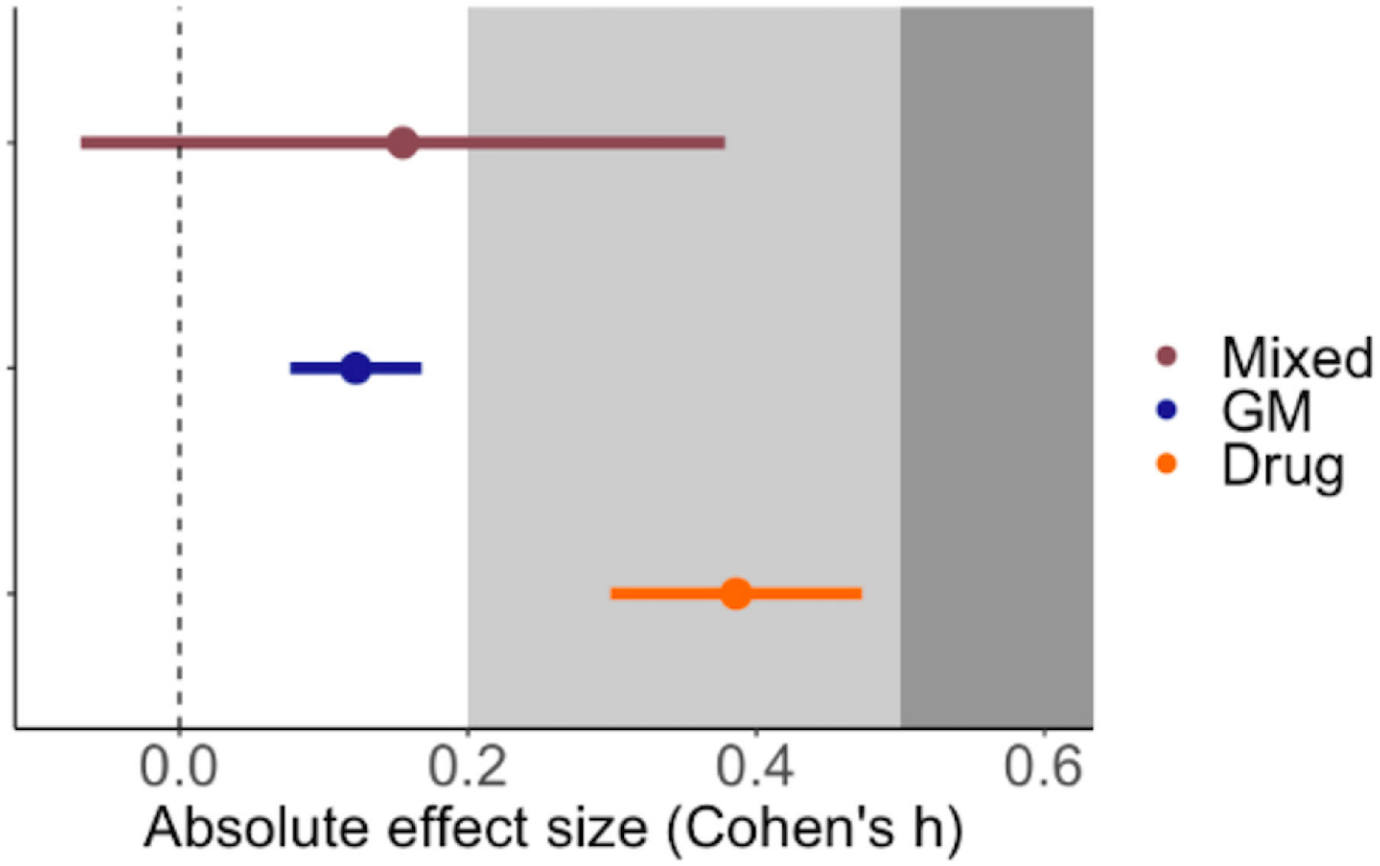
Mean ± SE effect size for the method of immune perturbation in the *P. berghei* model. GM = genetic modification (N = 13), Drug = agonist or antagonist chemical intervention (N = 13) and Mixed = any combination of two or more techniques from genetic modification, drug administration, surgery and adoptive cell transfer (N = 3). The grey shading denotes the reference for Cohen’s effect size bands: small (0.2) and medium (0.5) (23).

Of the four factors describing different aspects of experimental approaches only the method of immune manipulation explained significant heterogeneity across studies. Drug-induced perturbations of the innate immune system (including the administration of recombinant cytokines or monoclonal antibodies as well as small inhibitor molecules) generated the largest impact (**Figure 5**); the average Cohen’s h was 0.386, which is considered a small to medium effect (23) and translates to a ~15% difference in parasitaemia between treatment and control groups at the peak of infection. Furthermore, the impact of drug-induced perturbations was significantly higher than genetic modification (GM) (χ^2^ = 10.4520, p-value = 0.0012) or experiments that combined more than one methodology (mixed, χ^2^ = 4.561, p-value = 0.0327). Note that it wasn’t possible to estimate the relative effect of adoptive cell transfer or surgery alone because too few studies used these techniques. Finally, we found no moderating effects of the sampling regime, route of infection, or infecting dose (**Table 1**).

### 3.4 Assessment of publication bias

As demonstrated by funnel plots and the regression and rank tests (**Supplementary Figure 1** and **Supplementary Table 3**) we detected little evidence of publication bias in *P. chabaudi*, *P. yoelii* lethal or *P. yoelii* non-lethal. As we found an indication of publication bias in the *P. berghei* dataset (**Supplementary Table 3**) we carried out a sensitivity analysis by removing the three articles that contributed most to asymmetry. Removing these studies made little difference to the overall mean effect size (**Supplementary Figure 2**) indicating that our findings are unlikely to be affected by publication bias.

## 4 Discussion

We present a broad-ranging meta-analysis of studies spanning four decades of experimental manipulation to identify the role of innate immunity in controlling parasite replication early in infection. Because some studies manipulate immune function in ways that might facilitate parasite replication, whereas others would be expected to decrease parasitaemia, we quantify effect sizes on the absolute scale, which reports the magnitude (not the direction) of the difference between control and treatment groups. Overall, we reveal effect sizes (Cohen’s h) that are considered small for all four datasets, which span three parasite species (**Figure 2**) (23). The effect sizes range from 0.1475 to 0.2321, which translate to a change in parasitaemia of roughly 7-12% between treatment and control groups at the peak of infection, with *P. berghei* at the upper end of this range. We also reveal that for *P. berghei* the impacts of immune perturbation are larger when young or female mice are used, and when drugs are administered to manipulate the innate immune system. Furthermore, we observe the greatest impact when effector molecules (or signalling molecules downstream of transcription factors) are targeted (translating to an ~18% difference in peak parasitaemia). In contrast, there is extremely low heterogeneity across the *P. chabaudi* and *P. yoelii* datasets, which means that variation between studies is better explained by stochasticity (due to small sample sizes) than by host factors or experimental approaches.

The small effect sizes across all datasets have several non-mutually exclusive explanations. They may reflect redundancy in a complex biological system; the innate immune response to malaria is underpinned by a complex network of signalling pathways with considerable functional resilience (31, 32). Thus, the innate immune system may compensate and partially restore function following perturbation (33). The observation that effect sizes are larger when effector molecules (the output of signalling networks) are disrupted is consistent with the expectation of greater functional redundancy in upstream events, including receptor binding and signalling. Similarly, the smaller effect sizes observed in older or genetically modified hosts may be due to compensatory mechanisms that can restore immune function through time (34). Thus, the effect sizes we measure may simply reflect the lower bound of the overall effect of innate immunity on parasite replication. However, redundancy and robustness cannot explain the non-significant effects when major aspects of the innate immune system are disrupted or ablated (e.g., following antibody-mediated depletion of the entire myeloid lineage). Thus, an alternative explanation is that innate immunity has only a minor impact on the rate of parasite replication in the first hours and days of infection. That is not to say that innate immune cells do not remove infected red blood cells and free merozoites, but rather that the efficiency of this process depends upon help from the adaptive immune system (including CD4^+^ T cells and antibodies), which occurs later in infection (35). In this scenario, the early innate immune response may primarily function to mobilise and recruit phagocytes and lymphocytes to the inflamed spleen to coordinate a response that can effectively kill and clear parasites (36). Malaria initially triggers interferon-stimulated inflammation and emergency myelopoiesis in the bone marrow (37, 38), a conserved innate response that is also induced by a viral infection, injury and trauma (39, 40). Thus, there is no reason to assume that this should be well adapted to early control of malaria parasites. Meta-analysis could establish the importance of such mobilisation using complex multivariate analysis that tests for an interaction between time, trafficking and parasite burden, but the small number of published studies with relevant data preclude this at present (**Supplementary Table 2**).

We detected low heterogeneity across studies, indicating that the majority of variability is attributable to stochastic variation due to small sample sizes (30). Underpowered studies with small sample sizes are common in *in vivo* animal studies for ethical and logistical considerations (41). However, meta-analyses of studies with small sample sizes are more likely to find low heterogeneity and hence their scope is limited to probe between-study variation (42). This means that we can have confidence in our generalised conclusion that manipulating innate immunity has a small effect on parasite infection dynamics but it does not necessarily preclude individual effector molecules from having a more substantial impact. To overcome this limitation, larger-scale experiments are urgently needed to identify causes of mixed results in the literature, particularly in the *P. chabaudi* and *P. yoelii* models where we detected only ~1% heterogeneity. This was an unexpected finding as we expected *P. chabaudi* and non-lethal *P. yoelii* would have the largest effect sizes because they generally lead to self-resolving infections. In contrast, heterogeneity was significantly higher for *P. berghei* (~10%), which is often considered to be a poor model for studies of host immunity; experimental cerebral malaria is a common outcome of infection, which is lethal at a relatively low parasitaemia. How do we explain this counter-intuitive finding? *P. berghei* is not used more often than other parasite species and *P. berghei* experiments do not have larger sample sizes (**Supplementary Table 2**), suggesting a biological explanation. Perhaps because *P. berghei* infections are generally shorter than *P. chabaudi* and *P. yoelii* infections, it is easier to observe the impact of innate immunity when measuring changes in peak parasitaemia. This parameter is less likely to be influenced by other host factors (such as red cell availability), which may play a more dominant role in longer infections that can reach a higher pathogen load. Nonetheless, it is important to note that in all cases, the effect of manipulating the innate immune system was surprisingly modest and while *P. berghei* might reveal a greater role for innate immunity in controlling parasite replication this model may not reflect malaria more broadly or provide a good model for human infections.

We did not detect a moderating effect of either the timing of sampling or the infectious dose on the impact of immune perturbations. An impact of timing was expected because the possible difference in parasitaemia between control and treatment groups is smaller at the lower boundary. For example, when parasitaemia is low early in infection there is less scope for this to be reduced by immune augmentation as compared to at a higher parasitaemia, which is reached later in infection. Fortunately, standard effect sizes, like Cohen’s h, allow for the examination of effects free from this constraint, making our inference about the timing of sampling robust (23). The lack of a dose effect was also unexpected because several mathematical models have previously reported links between the initial parasite density (dose) and immune response to malaria (43–45). Nevertheless, infecting dose does not influence disease severity (46) and perhaps by assuming a strong role for the innate immune response in parasite control the field has overlooked the importance of disease tolerance mechanisms (38).

We did, however, identify host sex and age as moderators of innate immunity in the *P. berghei* model; immune perturbations had a greater impact in young and female mice (**Figure 3**). This relationship between host sex and innate immunity is consistent with findings that male mice are comparatively immunocompromised (and therefore more susceptible to infection) due to higher testosterone production (47). Yet the role of age in influencing mechanisms of host resistance remains an open question. Immune senescence (leading to reduced functionality) and chronic inflammation (so-called inflammaging) are both hallmarks of the ageing immune system, which could accelerate or slow parasite replication, respectively (48). Nonetheless, it is important to note that our study only includes sexually mature mice (between 5.5 to 11 weeks) and more experimental data are needed to directly examine the impact of innate immunity in older (as well as juvenile and neonatal) mice. As has been remarked elsewhere (49), we noticed that the reporting of host traits tends to be overlooked. For example, many studies in our dataset did not explicitly report host sex (n = 78 out of 196 studies), which means a direct comparison between male and female mice was not possible. Furthermore, the majority of studies used one strain (C57BL/6), constraining a full examination of host genetic background. However, the functional responses of C57BL/6 mice to *P. chabaudi* are typical of the responses of other commonly used inbred mouse strains (50) and consistent with our finding that responses of C57BL/6 mice to *P. berghei* do not significantly deviate from the average of all other combined strains. Nonetheless, the diversity of host strains used should be increased to provide the fine resolution required to comprehensively address the impact of varying host genetic backgrounds.

In addition to better reporting of host traits, we also advocate for improvements in data reporting. Parasitaemia (the percentage of infected RBC) is widely used as a proxy for parasite burden. However, this metric is problematic for several reasons. Most importantly, the proportion of infected RBC is confounded by the number of circulating RBC, which decreases as infection progresses. Parasites exploit and deplete RBC, and innate immune mechanisms clear RBC (including uninfected cells) and suppress RBC production (51). Thus, longitudinal parasitaemia data provide incomplete information on parasite burden and rates of replication, and it is impossible to distinguish whether top-down (e.g., parasite clearance) or bottom-up (e.g., red blood cell availability) mechanisms underlie the observed patterns. Addressing this limitation can be achieved by including RBC counts in addition to parasitaemia at each sampling point. While this is not routine, there is precedent to provide a fuller picture of infection dynamics; among the *P. chabaudi* studies in our dataset, all but 4 articles reported paired parasitaemia and RBC counts.

Additional insight is also likely to be gained by diversifying the hosts used to assess innate immunity to malaria. All but one study used inbred laboratory mice and the majority were housed under Specific Pathogen Free (SPF) conditions. Standard laboratory mouse husbandry has profound effects on the development of the immune system by reducing the diversity of the microbiota and eliminating natural exposure to pathogens, which continuously shape and remodel immune cell function (52, 53). For example, wild mice have a unique subset and modified tissue distribution of myeloid cells, which alters their innate response to microbial products (such as the TLR agonists CpG or LPS) (54). Caution is therefore required when translating findings from inbred laboratory mice to more ecologically realistic animal models and the human immune system.

Finally, we limited our meta-analysis to studies that infected mice by direct blood challenge, yet innate immunity might differ when infections are initiated by mosquito bite. Our previous work has shown that vector transmission resets parasite gene expression, which has a profound impact on the interaction between blood-stage parasites and the immune system (55). However, pre-erythrocytic parasites had minimal impact on the host response to blood-stage infection. Moreover, we have directly shown that the innate immune response to *P. falciparum* is comparable in naïve human hosts infected by mosquito bite versus blood challenge (56). We, therefore, have confidence that our conclusions will hold true when examined in the context of mosquito transmission, although additional experimental evidence will be required. The design of large-scale experiments that interrupt critical innate signalling pathways in multiple host strains infected by mosquito bite is, therefore, a priority. Large samples are required to allow the analysis of heterogeneity and also to overcome noise generated by variable and unknown infective doses. *Plasmodium chabaudi* is best suited to this task because genetically diverse strains with a broad spectrum of virulence after mosquito transmission are available (57).

## 5 Conclusions

Our meta-analysis summarized four decades of research to probe the effect of innate immunity manipulation in rodent models of malaria. We detected a small overall effect of perturbing the innate immune system on infection dynamics and revealed that manipulating effector molecules in young female mice by administering drugs (including monoclonal antibodies) has the largest impact on the replication of *P. berghei*. However, small sample sizes likely explain much of the variation across studies in the *P. chabaudi* and *P. yoelii* models. Why the impact of different experimental approaches varies between parasite species is unclear but raises questions about how to translate findings from rodent models to human malaria. Important differences exist between parasite species that infect humans and rodents, including that rodent *Plasmodium* spp. complete asexual replication in half the time it takes for the major human parasites (*P. falciparum* and *P. vivax)*. Perhaps the capacity for rodent parasites to replicate to much higher densities causes resource limitation to play a larger role in limiting parasite burden. No study system perfectly marries within-host ecological realism, translational value and tractability. Yet perhaps the rapid development of human challenge models will allow for the role of innate immunity early in infection to be tested experimentally by integrating human data with mathematical modelling to test long-standing (and alternative) hypotheses. Certainly, combining a more integrative approach with larger-scale experiments that report more comprehensive data will help resolve conflicting reports on the role of innate immunity in malaria.

## Data availability statement

The original contributions presented in the study are included in the article/**Supplementary Material**. Further inquiries can be directed to the corresponding author.

## Author contributions

Conceptualization, all authors; Analysis, AHM and TK; Writing-Original Draft, AHM, TK, SR; Writing-Review and Editing, all authors; Supervision, TK, SR.
