# Supplementary material for: The impact of innate immunity on malaria parasite infection dynamics in rodent models
Source: Front Immunol. 2023 Aug 14;14:1171176. doi: 10.3389/fimmu.2023.1171176 (PMC10461630; doi:10.3389/fimmu.2023.1171176)
Supplement: Supplementary file 1 [file DataSheet_1.pdf]

Supplementary Material

The impact of innate immunity on malaria parasite infection dynamics in rodent models

Alejandra Herbert-Mainero[1]*, Philip J. Spence[2], Sarah E. Reece[1,2†] & Tsukushi Kamiya[1,3,4†]

*Correspondence: a.herbert.mainero@utah.edu

†These authors contributed equally to this work and share senior authorship

Supplementary Figures

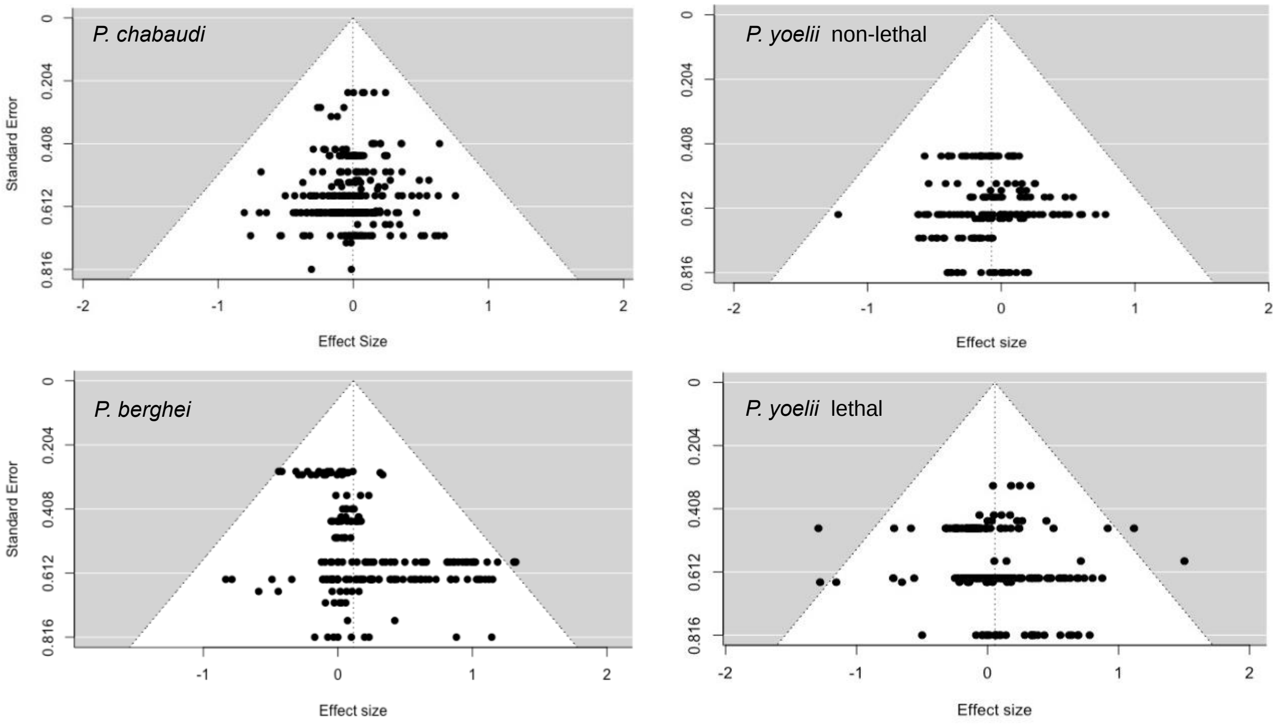

Supplementary Figure 1. Funnel plots showing the standard error (y-axis) against raw effect size (Cohen's; x-axis). The distribution of studies with high precision (near the top) is expected to be close to the average in the absence of publication bias, while those with low precision (near the

bottom) are expected to be distributed more widely on both sides. The grey area indicates a significant departure from this expectation with a 95% confidence level.

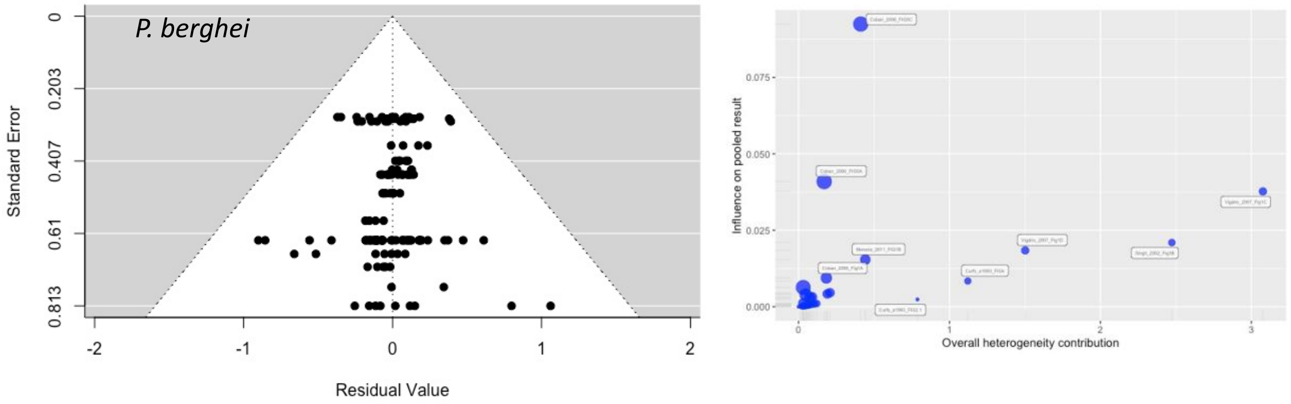

Supplementary Figure 2. Modified funnel plot of *P. berghei* data after removing three influential articles (left). "Boujat" graph showing mean effect size per study (right); the three articles removed correspond to the articles with the highest influence and contribution to heterogeneity. After omitting these articles, regression to inverse variance is not significant (Egger's test, intercept = 0.1038, se=0.0854, t=1.2151, df=127, p=0.2266, 95%CI -0.0652 -0.2728).

Supplementary Table 1. Explicit exclusion criteria. Table displays the eligibility criteria employed based on PICO defined elements (McKenzie et al., 2022).

	Explicit exclusion criteria
Subject	The subject is not related to the question of how innate immune components impact on rodent *Plasmodium* parasite burden
	Indirectly testing the effect: correlative or speculative mechanistic link to immune function
	Experiments have no control and treatment groups (for example host strains labelled as susceptible vs resistant)
Type	Not a primary research article (review, opinion, protocol, conference report)
	Article used data from another in the primary literature
	Not in English
Comparator	Uses parasite species that is not one of *P. berghei, yoelii, chabaudi, vinckei*
	Uses a non-mouse host species
	Infections initiated from a phase other than the blood stage (liver or mosquito derived infections)
	Co-infected or multiply infected hosts
	Uses genetically modified parasites (GMO) with a replication rate phenotype, or a mix of parasite antigens that activate the immune response, or attenuated parasites
	Uses hosts previously challenged with *Plasmodium* parasites or their antigens
Intervention	Data collected from *in vitro* or *ex vivo* approaches
	Not directly testing a component(s) of the innate immune response (e.g., by perturbing adaptive immune function)
Response	No quantification of parasite dynamics during infections (e.g., only point estimate of difference in parasite performance between treatment and

control). Cannot distinguish individual measurements from each treatment group

Day post infection information not included for parasite performance estimates

Data collected after the perturbation is expected to have exerted its influence on parasite performance

No justification given to relate how the sampling regime captures parasite dynamics of control and treatment groups

Data not suitable to relate to parasitaemia

Supplementary Table 2. The table shows the number of studies per moderator variables. Labels for levels within moderators: position in signaling network (in = input, out = output), cytokines/chemokines or their receptors (cc = cytokines/chemokines, rec = receptors), effector function (inflam = inflammatory, reg = regulatory, traff = cell trafficking), method of immune manipulation (GM = genetic modification) and route of infection (Ip = intraperitoneal injection, Iv = intravenous injection).

	Immune factors				Methodology		Host factors	
Parasite	Position in signalling network	Cytokines/ chemokines or their receptors	Effector function	Cell lineage	Method of immune manipulation	Route of infection	Sex	Age
	66	42	66	79	84	60	84	68
			37 inflam	15 lymphoid	7 mixed			
	31 in	28 cc	26 reg	21 myeloid	32 drug	47 Ip	5 male	
Pc	35 out	14 rec	3 traff	43 both	45 GM	13 Iv	26 female	NA
	24	11	29	31	29	28	32	20
	19 in	10 cc	16 inflam	1 lymphoid	3 mixed	25 Ip	1 male	
Pb	5 out	1 rec	12 reg	7 myeloid	13 drug	3 Iv	7 female	NA

			1 traff	23 both	13 GM			
PyL	24<br>14 in<br>10 out	13<br>10 cc<br>3 rec	22<br>14 inflam<br>4 reg<br>4 traff	28<br>3 lymphoid<br>2 myeloid<br>23 both	28<br>1 mixed<br>12 drug<br>15 GM	27<br>8 Ip<br>19 Iv	28<br>3 male<br>20 female	27<br>NA
PyNL	21<br>13 in<br>9 out	10<br>5 cc<br>5 rec	21<br>10 inflam<br>11 reg	29<br>3 lymphoid<br>7 myeloid<br>19 both	30<br>3 mixed<br>15 drug<br>12 GM	28<br>17 Ip<br>11 Iv	30<br>1 male<br>7 female	23<br>NA

Supplementary Table 3. Two common meta-analytic tests were conducted to assess publication bias (Rank tests and Egger’s test).

	Rank correlation test for funnel plot asymmetry	Egger’s test (regression to inverse variance)
P. chabaudi	Kendall's tau= = -0.0106, p-value = 0.7914	Intercept=-0.0216, SE=0.0460, t=-0.4697, df326, p-value=0.6389
P. berghei	Kendall's tau=0.2051, p-value < .0001	Intercept=0.2747, SE=0.1011, t=2.7180, df188, p-value=0.0072
P. yoelii lethal	Kendall's tau = 0.1900, p-value = 0.0002	Intercept=0.0915, SE=0.1163, t=0.3874, df224, p-value=0.4319
P. yoelii non-lethal	Kendall's tau= -0.0283, p-value = 0.5977	Intercept=0.0038, SE=0.1258, t=0.0308, df182, p-value=0.9754

Supplementary References

List of articles contributing data to the meta-analyses. Associated effect sizes and metadata can be found in Supplementary Data 1.

1. Abo T, Sekikawa H. Extrathymic T cells in malaria protection, including evidence for the onset of erythropoiesis in the liver during infection. *Arch. Histol. Cytol*. 2002;65(2):127–132. doi.org/10.1679/aohc.65.127

2. Bakir HY, Tomiyama-Miyaji C, Watanabe H, Nagura T, Kawamura T, Sekikawa H, Abo T. Reasons why DBA/2 mice are resistant to malarial infection: expansion of CD3int B220+ γδ T cells with double-negative CD4–CD8– phenotype in the liver. *Immunology*. 2006;117:127–135. doi.org/10.1111/j.1365-2567.2005.02273.x

3. Bastos KR, Barboza R, Elias RM, Sardinha LR, Grisotto MG, Marinho CR, et al. Impaired macrophage responses may contribute to exacerbation of blood-stage *Plasmodium chabaudi chabaudi* malaria in interleukin-12-deficient mice. *J. Interferon Cytokine Res*. 2002;22(12):1191–1199. doi.org/10.1089/10799900260475713

4. Batchelder JM, Burns JM Jr, Cigel FK, Lieberg H, Manning DD, Pepper BJ, et al. *Plasmodium chabaudi adami*: interferon-gamma but not IL-2 is essential for the expression of cell-mediated immunity against blood-stage parasites in mice. *Exp. Parasitol*. 2003;105(2):159–166. doi.org/10.1016/j.exppara.2003.12.003

5. Borges da Silva H, Fonseca R, Cassado A dos A, Machado de Salles É, de Menezes MN, Langhorne J, et al. In vivo approaches reveal a key role for DCs in CD4+ T cell activation and parasite clearance during the acute phase of experimental blood-stage malaria. *PLOS Pathog*. 2015;11(2):e1004598. doi.org/10.1371/journal.ppat.1004598

6. Campanella GS, Tager AM, El Khoury JK, Thomas SY, Abrazinski TA, Manice LA, et al. Chemokine receptor CXCR3 and its ligands CXCL9 and CXCL10 are required for the development of murine cerebral malaria. *Proc. Natl. Acad. Sci.* U.S.A. 2008;105(12):4814–4819. doi.org/10.1073/pnas.0801544105

7. Choudhury HR, Sheikh NA, Bancroft GJ, Katz DR, De Souza JB. Early nonspecific immune responses and immunity to blood-stage nonlethal *Plasmodium* yoelii malaria. *Infect. Immun*. 2000;68(11):6127–6132. doi.org/10.1128/IAI.68.11.6127-6132.2000

8. Clark IA, Hunt NH. Evidence for reactive oxygen intermediates causing hemolysis and parasite death in malaria. *Infect. Immun*. 1983;39(1):1–6. doi.org/10.1128/iai.39.1.1-6.1983

9. Clark IA, Hunt NH, Butcher GA, Cowden WB. Inhibition of murine malaria (*Plasmodium chabaudi*) in vivo by recombinant interferon-gamma or tumor necrosis factor, and its enhancement by butylated hydroxyanisole. *J. Immunol*. 1987;139(10):3493-6.

10. Coban C, Ishii KJ, Uematsu S, Arisue N, Sato S, Yamamoto M,et al. Pathological role of Toll-like receptor signaling in cerebral malaria. *Int. Immunol*. 2007;19(1):67-79. doi.org/10.1093/intimm/dxl123

11. Couper KN, Blount DG, Hafalla JC, van Rooijen N, de Souza JB, Riley EM. Macrophage-mediated but gamma interferon-independent innate immune responses control the primary wave of *Plasmodium yoelii* parasitemia. *Infect. Immun.* 2007;75(12):5806-5818. doi.org/10.1128/IAI.01005-07

12. Curfs JH, Hermsen CC, Kremsner P, Neifer S, Meuwissen JH, Van Rooyen N, Eling WM. Tumour necrosis factor-alpha and macrophages in *Plasmodium berghei*-induced cerebral malaria. *Parasitology*. 1993;107(Pt 2):125-134. doi.org/10.1017/s0031182000067226

13. Edwards CL, Best SE, Gun SY, Claser C, James KR, de Oca MM, Sebina I, Rivera Fde L, Amante FH, Hertzog PJ, Engwerda CR, Renia L, Haque A. Spatiotemporal requirements for IRF7 in mediating type I IFN-dependent susceptibility to blood-stage *Plasmodium* infection. *Eur. J. Immunol.* 2015;45(1):130-141. https://doi.org/10.1002/eji.201444824

14. Elased KM, Taverne J, Playfair JH. Malaria, blood glucose, and the role of tumour necrosis factor (TNF) in mice. *Clin. Exp. Immunol.*1996;105(3):443-449. doi.org/10.1046/j.1365-2249.1996.d01-781.x

15. Fontana MF, Baccarella A, Craft JF, Boyle MJ, McIntyre TI, Wood MD, Thorn KS, Lin L, Wohlfert E, Tallquist MD, Pepper M. A novel model of asymptomatic *Plasmodium* parasitemia that recapitulates elements of the human immune response to chronic infection. *PLOS ONE*. 2016;11(9):e0162132. doi.org/10.1371/journal.pone.0162132

16. Franklin BS, Rodrigues SO, Antonelli LR, Oliveira RV, Goncalves AM, Sales-Junior PA, Valente EP, Alvarez-Leite JI, Ropert C, Golenbock DT, Gazzinelli RT. MyD88-dependent activation of dendritic cells and CD4(+) T lymphocytes mediates symptoms, but is not required for the immunological control of parasites during rodent malaria. *Microbes Infect.* 2007;9(7):881-90.

17. Geurts N, Martens E, Verhenne S, Lays N, Thijs G, Magez S, et al. Insufficiently defined genetic background confounds phenotypes in transgenic studies as exemplified by malaria infection in Tlr9 knockout mice. *PLOS ONE*. 2011;6(11):e27131. doi.org/10.1371/journal.pone.0027131

18. Gowda NM, Wu X, Gowda DC. TLR9 and MyD88 are crucial for the development of protective immunity to malaria. *J. Immunol.* 2012;188(10):5073-85. doi.org/10.4049/jimmunol.1102143

19. Gramaglia I, Velez J, Combes V, Grau GE, Wree M, van der Heyde HC. Platelets activate a Pathogenic response to blood-stage *Plasmodium* infection but not a protective immune response. *Blood.* 2017;129(12):1669-79. doi.org/10.1182/blood-2016-08-733519

20. Hafalla JC, Burgold J, Dorhoi A, Gross O, Ruland J, Kaufmann SH, et al. Experimental cerebral malaria develops independently of caspase recruitment domain-containing protein 9 signaling. *Infect Immun*. 2012;80(3):1274-9. doi.org/10.1128/IAI.06033-11

21. Hahn WO, Butler NS, Lindner SE, Akilesh HM, Sather DN, Kappe SH, et al. cGAS-mediated control of blood-stage malaria promotes *Plasmodium*-specific germinal center responses. *JCI Insight*. 2018;3(2):e94142. doi.org/10.1172/jci.insight.94142

22. Haque A, Best SE, Ammerdorffer A, Desbarrieres L, de Oca MM, Amante FH, et al. Type I interferons suppress CD4, T-cell-dependent parasite control during blood-stage *Plasmodium* infection. *Eur. J. Immunol*. 2011;41(9):2688-98. doi.org/10.1002/eji.201141539

23. Hernandez-Valladares M, Naessens J, Musoke AJ, Sekikawa K, Rihet P, Ole-Moiyoi OK, et al. Pathology of Tnf-deficient mice infected with *Plasmodium chabaudi adami* 408XZ. *Exp Parasitol*. 2006;114(4):271-8. doi.org/10.1016/j.exppara.2006.04.003

24. Hou N, Zou Y, Piao X, Liu S, Wang L, Li S, et al. T-cell immunoglobulin- and mucin-domain-containing molecule 3 signaling blockade improves cell-mediated immunity against malaria. J. *Infect. Dis*. 2016;214(10):1547-56. doi.org/10.1093/infdis/jiw428

25. Ing R, Stevenson MM. Dendritic cell and NK cell reciprocal cross talk promotes gamma interferon-dependent immunity to blood-stage *Plasmodium chabaudi* AS infection in mice. *Infect Immun.* 2009;77(2):770-782. doi:10.1128/IAI.00994-08

26. Ing R, Gros P, Stevenson MM. Interleukin-15 enhances innate and adaptive immune responses to blood-stage malaria infection in mice. *Infect Immun.* 2005;73(5):3172-3177. doi:10.1128/IAI.73.5.3172-3177.2005

27. Jacobs P, Radzioch D, Stevenson MM. Nitric oxide expression in the spleen, but not in the liver, correlates with resistance to blood-stage malaria in mice. *J. Immunol.* 1995;155(11):5306-5313.

28. James KR, Soon MSF, Sebina I, et al. IFN Regulatory Factor 3 balances Th1 and T Follicular helper immunity during nonlethal blood-stage *Plasmodium* infection. *J. Immunol.* 2018;200(4):1443-1456. doi:10.4049/jimmunol.1700782

29. Kain KC, Lu Z, Finney CAM, Philpott DJ, LeBourhis L. Disruption of Nod-like Receptors alters inflammatory response to infection but does not confer protection in experimental cerebral malaria. *Am. J. Trop. Med. Hyg*. 2009;80(5):718-722. doi:10.4269/ajtmh.2009.80.718

30. Kim CC, Nelson CS, Wilson EB, Hou B, DeFranco AL, DeRisi JL. Splenic red pulp macrophages produce type I interferons as early sentinels of malaria infection but are dispensable for control. *PLOS ONE*. 2012;7(10):e48126. doi:10.1371/journal.pone.0048126

31. Kobayashi F, Ishida H, Matsui T, Tsuji M. Effects of in vivo administration of anti-IL-10 or anti-IFN-gamma monoclonal antibody on the host defense mechanism against *Plasmodium yoelii yoelii* infection. *J. Vet. Med. Sci*. 2000;62(6):583-587. doi:10.1292/jvms.62.583

32. Lacerda-Queiroz N, Riteau N, Eastman RT, et al. Mechanism of splenic cell death and host mortality in a *Plasmodium yoelii* malaria model. *Sci Rep.* 2017;7(1):10438. doi:10.1038/s41598-017-10776-2

33. Li C, Mannoor K, Inafuku M, et al. Protective function of an unconventional γδ T cell subset against malaria infection in apoptosis inhibitor deficient mice. *Cell Immunol*. 2012;279(2):151-159. doi:10.1016/j.cellimm.2012.09.012

34. Maglinao M, Klopfleisch R, Seeberger PH, Lepenies B. The C-type lectin receptor DCIR is crucial for the development of experimental cerebral malaria. *J. Immunol*. 2013;191(5):2551-2559

35. Mannoor MK, Weerasinghe A, Halder RC, Reza S, Morshed M, Ariyasinghe A, Watanabe H, Sekikawa H, Abo T. Resistance to malarial infection is achieved by the cooperation of NK1.1(+) and NK1.1(-) subsets of intermediate TCR cells which are constituents of innate immunity. *Cell Immunol.* 2001;211(2):96–104. doi:10.1006/cimm.2001.1833

36. Mastelic B, do Rosario AP, Veldhoen M, Renauld JC, Jarra W, Sponaas AM, Roetynck S, Stockinger B, Langhorne J. IL-22 protects against liver pathology and lethality of an experimental blood-stage malaria infection. *Front. Immunol.* 2012;3:85. doi:10.3389/fimmu.2012.00085

37. Muxel SM, Freitas do Rosário AP, Zago CA, Castillo-Méndez SI, Sardinha LR, Rodriguez-Málaga SM, Câmara NO, Álvarez JM, Lima MR. The spleen CD4+ T cell response to blood-stage *Plasmodium chabaudi* malaria develops in two phases characterized by different properties. *PLOS ONE.* 2011;6(7):e22434. doi:10.1371/journal.pone.0022434

38. Okada H, Suzue K, Imai T, Taniguchi T, Shimokawa C, Onishi R, Hirata J, Hisaeda H. A transient resistance to blood-stage malaria in interferon-γ-deficient mice through impaired production of the host cells preferred by malaria parasites. *Front. Microbiol.* 2015;6:600. doi:10.3389/fmicb.2015.00600

39. Patel SN, Lu Z, Ayi K, Serghides L, Gowda DC, Kain KC. Disruption of CD36 impairs cytokine response to *Plasmodium falciparum* glycosylphosphatidylinositol and confers susceptibility to severe and fatal malaria in vivo. *J. Immunol.* 2007;178(6):3954–3961. doi:10.4049/jimmunol.178.6.3954

40. Pattaradilokrat S, Li J, Wu J, Qi Y, Eastman RT, Zilversmit M, Nair SC, Huaman MC, Quinones M, Jiang H, Li N, Zhu J, Zhao K, Kaneko O, Long CA, Su XZ. *Plasmodium* genetic loci linked to host cytokine and chemokine responses. *Genes Immun.* 2014;15(3):145–152. doi:10.1038/gene.2013.74

41. Playfair JH. Lethal *Plasmodium yoelii* malaria: the role of macrophages in normal and immunized mice. *Bull World Health Organ*. 1979;57 Suppl 1:245-246.

42. Rudin W, Eugster HP, Bordmann G, Bonato J, Müller M, Yamage M, Ryffel B. Resistance to cerebral malaria in tumor necrosis factor-alpha/beta-deficient mice is associated with a reduction of intercellular adhesion molecule-1 up-regulation and T helper type I response. *Am. J. Pathol.* 1997;150(1):257-66

43. Sebina, I., James, K. R., Soon, M. S., Fogg, L. G., Best, S. E., Labastida Rivera, F.,et al. IFNAR1-Signalling obstructs ICOS-mediated humoral immunity during non-lethal blood-stage *Plasmodium* infection. *PLOS Pathog*. 2016;12(11), e1005999. doi.org/10.1371/journal.ppat.1005999.

44. Sellau J, Alvarado CF, Hoenow S, Mackroth MS, Kleinschmidt D, Huber S, Jacobs T. IL-22 dampens the T cell response in experimental malaria. *Sci. Rep.* 2016;6:28058. doi.org/10.1038/srep28058.

45. Serghides L, Patel SN, Ayi K, Lu Z, Gowda DC, Liles WC, Kain KC. Rosiglitazone modulates the innate immune response to *Plasmodium* falciparum infection and improves outcome in experimental cerebral malaria. *J. Infect. Dis.* 2009;199(10):1536–1545. doi.org/10.1086/598222.

46. Singh RP, Kashiwamura S, Rao P, Okamura H, Mukherjee A, Chauhan VS. The role of IL-18 in blood-stage immunity against murine malaria *Plasmodium yoelii* 265 and *Plasmodium berghei* ANKA. *J. Immunol.* 2002;168(9):4674–4681. doi.org/10.4049/jimmunol.168.9.4674.

47. Spaulding E, Fooksman D, Moore JM, Saidi A, Feintuch CM, Reizis B, Chorro L, Daily J, Lauvau G. STING-Licensed macrophages prime type I IFN production by plasmacytoid dendritic cells in the bone marrow during severe *Plasmodium yoelii* malaria. *PLOS Pathog.* 2016;12(10):e1005975. doi.org/10.1371/journal.ppat.1005975.

48. Sponaas AM, Freitas do Rosario AP, Voisine C, Mastelic B, Thompson J, Koernig S, Jarra W, et al. Migrating monocytes recruited to the spleen play an important role in control of blood stage malaria. *Blood.* 2009;114(27):5522–5531. doi.org/10.1182/blood-2009-04-217489.

49. Stevenson MM, Ghadirian E. Human recombinant tumor necrosis factor alpha protects susceptible A/J mice against lethal *Plasmodium chabaudi* AS infection. *Infect Immun.* 1989;57(12):3936–3939. doi.org/10.1128/iai.57.12.3936-3939.1989.

50. Stevenson, Mary M, Mi Fong Tam, and Darlene Rae. Dependence on cell-mediated mechanisms for the appearance of Crisis Forms during *Plasmodium chabaudi* AS Infection in C57BL/6 Mice." *Microb. Pathog* 1990; 9(5):303–14. doi.org/10.1016/0882-4010(90)90065-x.

51. Stevenson MM, Ghadirian E, Phillips NC, Rae D, Podoba JE. Role of mononuclear phagocytes in elimination of *Plasmodium chabaudi* AS infection. *Parasite Immunol.* 1989;11(5):529–544. doi.org/10.1111/j.1365-3024.1989.tb00687.x.

52. Stevenson MM, Tam MF, Nowotarski M. Role of interferon-gamma and tumor necrosis factor in host resistance to *Plasmodium chabaudi* AS. *Immunol. Lett.* 1990;25(1-3):115–121. doi.org/10.1016/0165-2478(90)90101-u

53. Stevenson MM, Tam MF, Wolf SF, Sher A. IL-12-induced protection against blood-stage *Plasmodium chabaudi* AS requires IFN-gamma and TNF-alpha and occurs via a nitric oxide-dependent mechanism. *J. Immunol.* 1995;155(5):2545–2556.

54. Su Z, Fortin A, Gros P, Stevenson MM. Opsonin-independent phagocytosis: An effector mechanism against acute blood-stage *Plasmodium chabaudi* AS infection. *J. Infect. Dis.* 2002;186(9):1321–1329. doi:10.1086/344576

55. Tamura T, Akbari M, Kimura K, Kimura D, Yui K. Flt3 ligand treatment modulates parasitemia during infection with rodent malaria parasites via MyD88- and IFN-gamma-dependent mechanisms. *Parasite Immunol.* 2014;36(2):87–99. doi:10.1111/pim.12085

56. Taverne J, Sheikh N, de Souza JB, Playfair JH, Probert L, Kollias G. Anaemia and resistance to malaria in transgenic mice expressing human tumour necrosis factor. *Immunology*. 1994;82(3):397–403.

57. Taverne J, Tavernier J, Fiers W, Playfair JH. Recombinant tumour necrosis factor inhibits malaria parasites in vivo but not in vitro. *Clin. Exp. Immunol*. 1987;67(1):1–4.

58. Theeß W, Sellau J, Steeg C, et al. Myeloperoxidase attenuates Pathogen clearance during *Plasmodium yoelii* nonlethal infection. *Infect. Immun*. 2016;85(1):e00475-16. doi:10.1128/IAI.00475-16

59. Tsutsui N, Kamiyama T. Suppression of in vitro IFN-gamma production by spleen cells of *Plasmodium chabaudi*-infected C57BL/10 mice exposed to dexamethasone at a low dose. *Int. J. Immunopharmacol.* 1998;20(4-5):141–152. doi:10.1016/s0192-0561(98)00019-8

60. Tsutsui N, Kamiyama T. Transforming growth factor beta-induced failure of resistance to infection with blood-stage *Plasmodium chabaudi* in mice. *Infect. Immun.* 1999;67(5):2306–2311. doi:10.1128/IAI.67.5.2306-2311.1999

61. Van der Heyde HC, Gu Y, Zhang Q, Sun G, Grisham MB. Nitric oxide is neither necessary nor sufficient for resolution of *Plasmodium chabaudi* malaria in mice. *J. Immunol.* 2000;165(6):3317–3323. doi:10.4049/jimmunol.165.6.3317

62. Vigário AM, Belnoue E, Grüner AC, Mauduit M, Kayibanda M, Deschemin JC,et al. Recombinant human IFN-alpha inhibits cerebral malaria and reduces parasite burden in mice. *J. Immunol*. 2007;178(10):6416-25. doi: 10.4049/jimmunol.178.10.6416.

63. Villegas-Mendez A, Inkson CA, Shaw TN, Strangward P, Couper KN. Long-Lived CD4+IFN-γ+ T cells rather than short-lived CD4+IFN-γ+IL-10+ T cells initiate rapid IL-10 production to suppress anamnestic T cell responses during secondary malaria infection. *J. Immunol*. 2016;197(8):3152-3164. doi: 10.4049/jimmunol.1600968.

64. Voisine C, Mastelic B, Sponaas AM, Langhorne J. Classical CD11c+ dendritic cells, not plasmacytoid dendritic cells, induce T cell responses to *Plasmodium chabaudi* malaria. *Int. J. Parasitol*. 2010;40(6):711-9. doi: 10.1016/j.ijpara.2009.11.005.

65. Weidanz WP, LaFleur G, Brown A, Burns JM Jr, Gramaglia I, van der Heyde HC. Gammadelta T cells but not NK cells are essential for cell-mediated immunity against *Plasmodium chabaudi* malaria. *Infect. Immun.* 2010 Oct;78(10):4331-40. doi: 10.1128/IAI.00539-10.

66. Weidanz WP, Lafleur G, Kita-Yarbro A, Nelson K, Burns JM Jr. Signalling through the IL-2 receptor γ(c) peptide (CD132) is essential for the expression of immunity to *Plasmodium chabaudi adami* blood-stage malaria. *Parasite Immunol.* 2011;33(9):512-6. doi: 10.1111/j.1365-3024.2011.01298.x.

67. Wu J, Xia L, Yao X, Yu X, Tumas KC, Sun W,et al.. The E3 ubiquitin ligase MARCH1 regulates antimalaria immunity through interferon signaling and T cell activation. *Proc. Natl. Acad. Sci.* U.S.A. 2020;117(28):16567-16578. doi:10.1073/pnas.2004332117.

68. Wu X, Dayanand KK, Thylur RP, Norbury CC, Gowda DC. Small molecule-based inhibition of MEK1/2 proteins dampens inflammatory responses to malaria, reduces parasite load, and mitigates Pathogenic outcomes. *J. Biol. Chem*. 2017;292(33):13615-13634. doi:10.1074/jbc.M116.770313.

69. Wu X, Thylur RP, Dayanand KK, Punnath K, Norbury CC, Gowda DC. IL-4 treatment mitigates experimental cerebral malaria by reducing parasitemia, dampening inflammation, and lessening the cytotoxicity of T cells. *J. Immunol.* 2021;206(1):118-131. doi:10.4049/jimmunol.2000779.

70. Wunderlich F, Dkhil MA, Mehnert LI, Braun JV, El-Khadragy M, Borsch E,et al.. Testosterone responsiveness of spleen and liver in female lymphotoxin beta receptor-deficient mice resistant to blood-stage malaria. *Microbes Infect*. 2005;7(3):399-409. doi: 10.1016/j.micinf.2004.11.016.

71. Yao X, Wu J, Lin M, Sun W, He X, Gowda C,et al.. Increased CD40 expression enhances early STING-Mediated Type I Interferon Response and host survival in a rodent malaria model. *PLOS Pathog*. 2016;12(10):e1005930. doi: 10.1371/journal.ppat.1005930.

72. Xia L, Wu J, Pattaradilokrat S, Tumas K, He X, Peng YC, Huang R, et al. Detection of Host Pathways Universally Inhibited after *Plasmodium yoelii* Infection for Immune Intervention. *Sci Rep*. 2018;8(1):15280. doi: 10.1038/s41598-018-33599-1.

73. Zander RA, Guthmiller JJ, Graham AC, Pope RL, Burke BE, Carr DJ, Butler NS. Type I Interferons induce T regulatory 1 responses and restrict humoral immunity during experimental malaria. *PLOS Pathog.* 2016;12(10):e1005945. doi: 10.1371/journal.ppat.1005945.

74. Zhang Y, Zhu X, Feng Y, Pang W, Qi Z, Cui L, Cao Y. TLR4 and TLR9 signals stimulate protective immunity against blood-stage *Plasmodium yoelii* infection in mice. *Exp Parasitol.* 2016;170:73-81. doi: 10.1016/j.exppara.2016.09.003.
